# Pausing before Surgery Referral in Patients with ESKD on Hemodialysis to Ensure Patient-Centered Care

**DOI:** 10.34067/KID.0000000731

**Published:** 2025-02-06

**Authors:** Amit Pujari, Theodore H. Yuo, Daniel Hall, Jane O. Schell

**Affiliations:** 1 Division of Vascular Surgery, University of Washington, Seattle, Washington; 2 Division of Vascular Surgery, University of Pittsburgh Medical Center, Pittsburgh, Pennsylvania; 3 Department of Surgery, University of Pittsburgh School of Medicine, Pittsburgh, Pennsylvania; 4 Section of Palliative Care and Medical Ethics, University of Pittsburgh School of Medicine, Pittsburgh, Pennsylvania; 5 Division of Renal Electrolyte, University of Pittsburgh School of Medicine, Pennsylvania

**Keywords:** dialysis, dialysis access, quality of life, palliative care

## Clinical Case

During a typical day rounding at an outpatient hemodialysis center, you notice Mr. S, a 75-year-old man with multiple comorbidities, receiving dialysis. Mr. S has congestive heart failure (CHF) and pulmonary hypertension from rheumatic heart disease; he has undergone mechanical aortic valve replacement. A recent exacerbation of CHF resulted in urgent initiation of hemodialysis through a tunneled dialysis catheter (TDC). He is unable to perform the self-care required for peritoneal dialysis. Given that Mr. S has transitioned to routine outpatient hemodialysis, the nurse manager approaches you regarding arteriovenous (AV) access as the next step.

Six weeks after dialysis initiation, he was referred to a vascular surgeon. He is nervous about undergoing more surgery, and he worries about spending too much time in the hospital. You emphasize the risk of catheter-related infections, which are mitigated with an AV fistula (AVF) or AV graft. The vascular surgery team sees the patient promptly in clinic and deems him appropriate for an upper arm AVF. Although the surgeon notes some concerning details in the patient's history, he is scheduled for surgery. After undergoing AVF creation, the patient's fistula fails to mature, and he is scheduled for additional surgeries. Eventually, the patient's fistula thromboses before it is ever accessed, and the patient's functional status declines with each subsequent surgery. Before his next scheduled fistula revision, Mr. S is hospitalized with heart failure exacerbation resulting in respiratory failure. After 10 days on a ventilator, his family elects to remove life support and transition care to comfort measures only.

## Background

Nephrology clinicians often care for patients who initiate hemodialysis urgently with a TDC placed in the setting of ARF that then progresses to chronic renal replacement therapy. For older patients with multiple comorbid conditions like Mr. S, survival is limited even with dialysis, especially within the first 120 days of initiation.^1^ For them, AV access creation can unintentionally lead to setbacks and complications. Recent studies underscore that conservative kidney management (CKM) may result in more time spent at home for elderly patients compared with those started on dialysis.^2^ There is a need for a patient-centered approach for patients with serious medical comorbidities.

Many patients start dialysis during an acute hospitalization without an informed understanding of their prognosis. The stress and chaos of deciding how to manage the kidney failure supersede meaningful goals-of-care (GOCs) discussions about how likely dialysis will achieve their goals.^3^ As a result, most patients elect a trial of hemodialysis with placement of a TDC.

For most patients, the expected next step is vascular surgery referral for access creation. Governmental organizations, such as the US Center for Medicare & Medicaid Services, set metrics evaluating the quality of care and reimbursement to dialysis centers with a focus on vascular access outcomes, such as rate of long-term catheter use. The unintended outcome of care processes focused on achieving a mature permanent access may neglect the question of how well the dialysis is being tolerated or whether it is achieving the hoped-for benefits.

Vascular access surgery can also add additional burdens. Although AV access surgery is often considered minor compared with other vascular surgeries, this characterization minimizes the physiologic changes incurred by creation of an AVF and the operative risks within this medically complex population.^4^ In addition, patients on dialysis are more likely to have a lower health-related quality of life and life expectancy than many other vascular patients.^5^ The 2019 Kidney Disease Outcomes Quality Initiative guidelines emphasize a more holistic framework like the ESKD Life-Plan.^6,7^ Yet in practice, vascular surgery evaluations are typically guided by optimal venous anatomy and duration of indwelling TDC.

Rather than automatic referral to a vascular surgeon for access creation, we present a framework that encourages nephrology providers to pause before referral for patients with serious illness and instead engage in a larger discussion of the patient's prognosis and preferences to guide access decisions and overall focus of care.

## A Patient-Centered Approach for Patients Initiating Dialysis

We propose that nephrologists pause to check-in on how dialysis is going, especially for those with serious illness, rather than a default referral to vascular surgery. These check-in conversations represent an opportunity to evaluate how hemodialysis is affecting a patient's life and whether it is helping to achieve their goals.

The danger associated with not using this pause is that high-risk patients will undergo surgeries that may diminish quality of life and increase risk of complications, especially in patients facing limited life expectancy. Vascular surgeons evaluating these seriously ill patients may assume values, and because GOCs have already been explored, they may therefore be reluctant to deviate from the established plan.

Vascular access surgery can be successful, but may be harmful when performed in patients whose values and perioperative risk have not been assessed.^8^ Even seemingly minor procedures can exacerbate underlying conditions that result in emergency department visits and hospitalizations.

Although all patients initiated on hemodialysis warrant a pause, patients with serious illness are more likely to benefit from this approach before referral to vascular surgery. Patient populations who warrant a check-in conversation include, but are not limited to, those with advanced age; extensive medical comorbidities, such as severe CHF, chronic obstructive pulmonary disease, coronary artery disease, or peripheral vascular disease; and those with frailty, functional dependence for activities of daily living, or dementia.^1^

## What Does This Conversation Look Like?

Ideally, the outpatient nephrologist is best suited to have this conversation. Nephrologists function as the leader of the multidisciplinary dialysis team and often serve as an essential primary care doctor for patients on dialysis. An ideal time for this conversation is during the initial care planning visit that all new outpatients on hemodialysis must complete within the first 90 days of dialysis.

There are methods for conducting GOC conversations that can be used to guide nephrology providers with limited palliative care training.^9^ A general framework for the conversation's key steps is included with examples below.

## Key Steps to a Patient-Centered Conversation with Example Phrases

1. Frame and ask permission to have the conversation
  - “You've been on dialysis for ‘X amount of time,’ can we take a moment to talk about how it has been going and what to expect going forward?”
2. Use open-ended questions that assess the patient's experience, understanding, values, and goals.
  - “How have things been going on dialysis?”
  - “What has surprised you about being on dialysis?”
  - “What are you hoping dialysis might help you do or achieve?”
  - “What concerns do you have about your life on dialysis in the future?”
3. Clarify any pertinent knowledge gaps or questions including relevant prognostic information specific to the patient's condition.
  - “We worry that at this point, your kidneys are unlikely to recover. It's important for us to talk about how dialysis is going and whether we should proceed with doing things like putting a permanent shunt in your arm to do long-term dialysis.”
4. Deliberate next steps based on the values and goals expressed.
  - Hemodialysis supports their goals and is tolerable.
  - Hemodialysis is proving to be burdensome, but allowing more meaningful time.
  - Hemodialysis does meet patient goals, or symptoms are debilitating.

For patients where dialysis supports their goals and allows an acceptable quality of life, referral for surgery is appropriate. Following the ESKD Life-Plan, optimal dialysis access can be determined. A hand-off discussion or email between the outpatient nephrologist and surgeon can also foster additional discussion to guide care plans.

For patients who find that dialysis is falling short of their expectations and negatively affecting their quality of life or for those who have an unacceptably high risk with even minor surgeries, a change in approach is needed. In these patients, creation of surgical AV access should be deferred, and hemodialysis can be continued through a catheter indefinitely.

For patients whose goals are not being met by dialysis and decide to stop dialysis, CKM is recommended. CKM may be comanaged by providers from nephrology, palliative care, and hospice care when appropriate. Palliative care providers are medical specialists who treat symptoms, provide psychosocial support, and facilitate complex medical decision-making that includes transitions to end-of-life care.

Ideally all patients started on hemodialysis could have ongoing check-in conversations to reflect on how hemodialysis is affecting their life; however, the practicality of this is limited by several time and structural constraints. Most nephrologists already have busy, overscheduled days, and providers are not incentivized to have good conversations. Currently, governmental institutions tend to track and measure disease-based metrics, such as dialysis dose, anemia, and access outcomes, rather than patient-based metrics, such as GOCs or advanced care planning. Soon, however, more patient-centered measures may be incorporated into quality metrics focused on patient priorities and experience.^10^

## Conclusion

Nephrologists should use initial follow-up encounters after urgent initiation of dialysis to check-in with patients regarding whether dialysis is supporting their GOCs. A pause at this point, before referral for surgical intervention, will enable clinicians to identify high-risk patients, clarify their preferences, and ensure care is consistent with their goals and preferences.

## Supplementary Material

SUPPLEMENTARY MATERIAL
