# Supplementary material for: Pausing before Surgery Referral in Patients with ESKD on Hemodialysis to Ensure Patient-Centered Care
Source: Kidney360. 2025 Feb 6;6(3):473–5. doi: 10.34067/KID.0000000731 (PMC11970846; doi:10.34067/KID.0000000731)
Supplement: SUPPLEMENTARY MATERIAL [file kidney360-6-473-s001.pdf]

ASN American Society of Nephrology · JASN Journal of the American Society of Nephrology · KIDNEY360 Accessing Our World From Every Angle · CJASN Clinical Journal of the American Society of Nephrology

ASN Journal Disclosure Form

As per ASN journal policy, I have disclosed any financial relationships or commitments I have held in the past 36 months as included below. I have listed my Current Employer below to indicate there is a relationship requiring disclosure. If no relationship exists, my Current Employer is not listed.

D. Hall reports the following:
Employer: University of Pittsburgh; and Other Interests or Relationships: Unpaid consultant for FutureAssure, LLC, a medical device company aiming to assess frailty.

I understand that the information above will be published within the journal article, if accepted, and that failure to comply and/or to accurately and completely report the potential financial conflicts of interest could lead to the following: 1) Prior to publication, article rejection, or 2) Post-publication, sanctions ranging from, but not limited to, issuing a correction, reporting the inaccurate information to the authors' institution, banning authors from submitting work to ASN journals for varying lengths of time, and/or retraction of the published work.

Name: Daniel E. Hall
Manuscript ID: K360-2024-000716R2
Manuscript Title: Taking a pause before surgery referral in End-Stage Kidney Disease patients on hemodialysis to ensure patient-centered care
Date of Completion: January 16, 2025
Disclosure Updated Date: January 16, 2025

ASN American Society of Nephrology
JASN Journal of the American Society of Nephrology
KIDNEY360 Accessing Our World From Every Angle
CJASN Clinical Journal of the American Society of Nephrology

ASN Journal Disclosure Form

As per ASN journal policy, I have disclosed any financial relationships or commitments I have held in the past 36 months as included below. I have listed my Current Employer below to indicate there is a relationship requiring disclosure. If no relationship exists, my Current Employer is not listed.

A. Pujari has nothing to disclose.

I understand that the information above will be published within the journal article, if accepted, and that failure to comply and/or to accurately and completely report the potential financial conflicts of interest could lead to the following: 1) Prior to publication, article rejection, or 2) Post-publication, sanctions ranging from, but not limited to, issuing a correction, reporting the inaccurate information to the authors' institution, banning authors from submitting work to ASN journals for varying lengths of time, and/or retraction of the published work.

Name: Amit Pujari
Manuscript ID: K360-2024-000716R2
Manuscript Title: Taking a pause before surgery referral in End-Stage Kidney Disease patients on hemodialysis to ensure patient-centered care
Date of Completion: February 1, 2025
Disclosure Updated Date: February 1, 2025

ASN American Society of Nephrology
JASN Journal of the American Society of Nephrology
KIDNEY360 Accessing Our World From Every Angle
CJASN Clinical Journal of the American Society of Nephrology

ASN Journal Disclosure Form

As per ASN journal policy, I have disclosed any financial relationships or commitments I have held in the past 36 months as included below. I have listed my Current Employer below to indicate there is a relationship requiring disclosure. If no relationship exists, my Current Employer is not listed.

J. Schell reports the following:
Employer: University of Pittsburgh Medical Center; and Honoraria: uptodate.

I understand that the information above will be published within the journal article, if accepted, and that failure to comply and/or to accurately and completely report the potential financial conflicts of interest could lead to the following: 1) Prior to publication, article rejection, or 2) Post-publication, sanctions ranging from, but not limited to, issuing a correction, reporting the inaccurate information to the authors' institution, banning authors from submitting work to ASN journals for varying lengths of time, and/or retraction of the published work.

Name: Jane O. Schell
Manuscript ID: K360-2024-000716R2
Manuscript Title: "Taking a pause before surgery referral in End-Stage Kidney Disease patients on hemodialysis to ensure patient-centered care"
Date of Completion: February 1, 2025
Disclosure Updated Date: March 28, 2024

ASN
American Society of Nephrology
JASN
Journal of the American Society of Nephrology
KIDNEY360
Accessing Our World From Every Angle
CJASN
Clinical Journal of the American Society of Nephrology

ASN Journal Disclosure Form

As per ASN journal policy, I have disclosed any financial relationships or commitments I have held in the past 36 months as included below. I have listed my Current Employer below to indicate there is a relationship requiring disclosure. If no relationship exists, my Current Employer is not listed.

T. Yuo reports the following:
Employer: University of Pittsburgh Medical Center; University of Pittsburgh Physicians; Consultancy: Merit Medical; Ownership Interest: Tesla, Palo Alto Networks, Broadcom,; Research Funding: Merit Medical; and Honoraria: Merit Medical.

I understand that the information above will be published within the journal article, if accepted, and that failure to comply and/or to accurately and completely report the potential financial conflicts of interest could lead to the following: 1) Prior to publication, article rejection, or 2) Post-publication, sanctions ranging from, but not limited to, issuing a correction, reporting the inaccurate information to the authors' institution, banning authors from submitting work to ASN journals for varying lengths of time, and/or retraction of the published work.

Name: Theodore H. Yuo
Manuscript ID: K360-2024-000716R2
Manuscript Title: Taking a pause before surgery referral in End-Stage Kidney Disease patients on hemodialysis to ensure patient-centered care
Date of Completion: February 2, 2025
Disclosure Updated Date: February 2, 2025
